# Beyond the Counter: Assessing NSAIDs Dispensing and Use Among Community Pharmacies in Saudi Arabia’s Eastern Region: A Cross-Sectional Study

**DOI:** 10.3390/pharmacy14050123

**Published:** 2026-08-20

**Authors:** Mohamed A. Albekery, Helal F Hetta, Shatha A. Almikhlal, Aisha Y. Alfraih, Nora Aldhuhayyan, Zahra Alarbsh, Abdulrahman Abdullah Alnijadi, Monther Alsultan, Abdullah Al Hamid

**Affiliations:** 1 Department of Pharmacy Practice, College of Clinical Pharmacy, King Faisal University, Al-Ahsa 31982, Saudi Arabia; sgg3468@gmail.com (S.A.A.); aishaalfraih3@gmail.com (A.Y.A.); noraadeldh@gmail.com (N.A.); zahra2001maher@hotmail.com (Z.A.); aalnijadi@kfu.edu.sa (A.A.A.); malsultan@kfu.edu.sa (M.A.); aalhamid@kfu.edu.sa (A.A.H.); 2 Division of Microbiology, Immunology and Biotechnology, Department of Natural Products and Alternative Medicine, Faculty of Pharmacy, University of Tabuk, Tabuk 71491, Saudi Arabia; hhussen@ut.edu.sa

**Keywords:** non-steroidal anti-inflammatory drugs (NSAIDs), drug utilization, community pharmacy, non-prescription dispensing, medication safety

## Abstract

**Background:** Non-steroidal anti-inflammatory drugs (NSAIDs) are widely used for pain, inflammation, and fever. Although many NSAIDs are available without a prescription, inappropriate use may increase the risk of renal, gastrointestinal, and cardiovascular complications. This study evaluated NSAID dispensing patterns and potential risk practices in community pharmacies in the Eastern Province of Saudi Arabia. **Methods:** A cross-sectional study was conducted in 90 community pharmacies across Al-Ahsa, Al-Dammam, and Jubail Industrial City between February and March 2025. Data on demographics, NSAID type, indication, dosage, frequency, duration, and concomitant medication use were collected and analyzed descriptively. **Results:** Among the 171 documented NSAID dispensing cases, 98 (57.3%) occurred without a prescription, whereas 73 (42.7%) involved prescription-based dispensing. Ibuprofen (62%) and diclofenac (36.3%) were the most commonly dispensed agents, with oral tablets being the preferred formulation (76.6%). Most documented treatment durations were short (3–7 days) with once- or twice-daily dosing. Common documented indications included dental conditions, pain, and inflammation, although the indication was not specified in 57.3% of cases. At least one potential interaction was identified in 11(7.8%, 95% CI 4.4–13.4%) of the 141 cases with concomitant medication use, corresponding to 13 (5.9%, 95% CI 3.5–9.9%) potential interaction pairs. **Conclusions:** Frequent non-prescription NSAID dispensing, incomplete documentation, and potentially clinically relevant drug interactions highlight the need to strengthen medication review, documentation, public awareness, regulatory oversight, and pharmacist counseling in community pharmacy practice.

## 1. Introduction

### 1.1. Background on NSAID Use

Non-steroidal anti-inflammatory drugs (NSAIDs) are among the most widely prescribed and consumed analgesics worldwide for managing pain, fever, and inflammatory conditions [1]. They are routinely used for rheumatoid arthritis, musculoskeletal injuries, dental pain, dysmenorrhea, and postoperative discomfort [2]. NSAIDs exert their therapeutic effects primarily by inhibiting cyclooxygenase (COX) enzymes, and they are broadly classified into non-selective COX-1/COX-2 inhibitors and selective COX-2 inhibitors.

Despite their proven efficacy, NSAIDs carry significant risk profiles. Gastrointestinal (GI) complications ranging from dyspepsia to ulceration and life-threatening hemorrhage occur in 1–4% of chronic users [3,4]. Furthermore, NSAIDs are strongly associated with nephrotoxicity (acute kidney injury and chronic kidney disease progression), hepatotoxicity, and adverse cardiovascular events such as myocardial infarction and stroke [5,6]. Because NSAIDs are available both via prescription and over the counter (OTC), easy access frequently leads to unsupervised consumption, inappropriate dosing, prolonged usage, and unmonitored polypharmacy [7].

### 1.2. Global Perspective on NSAID Utilization

Globally, non-prescription NSAID use accounts for a substantial proportion of overall pain management, driven by affordability and a high level of public perception regarding their safety [7,8]. The international literature consistently reports that over half of NSAID consumers self-medicate without prior clinical consultation [8,9]. This widespread self-administration, combined with limited consumer awareness of contraindications, continues to drive preventable hospital admissions and healthcare expenditures globally [9,10]. Consequently, health authorities emphasize the importance of continuous monitoring and active pharmacist oversight to mitigate preventable adverse drug reactions (ADRs).

### 1.3. NSAID Use in the Saudi Context

In Saudi Arabia, NSAIDs are readily accessible as non-prescription medications in community pharmacies. National surveys indicate high rates of self-medication, with approximately 67% of individuals consuming NSAIDs without medical guidance [8]. Public awareness regarding potential adverse outcomes remains suboptimal; for instance, studies show that less than half (45.2%) of consumers recognize the link between NSAIDs and peptic ulcer disease or renal impairment [8,11].

While the existing Saudi literature has established the high prevalence of consumer self-medication and baseline public knowledge gaps, these studies have predominantly focused on patient-reported questionnaires or broad national surveys. Consequently, critical pharmacy-level operational metrics such as actual point-of-sale dispensing habits, patient risk screening rigor, counseling thoroughness, and documentation practices remain under-explored in regional community settings [12].

### 1.4. Role of Community Pharmacies

Community pharmacists represent the primary gatekeepers for non-prescription NSAID supply and are uniquely positioned to intervene against inappropriate use. Pharmacists are expected to assess patient risk factors (e.g., age, renal function, history of GI ulcers, concurrent anticoagulants), recommend appropriate dosages, and counsel on adverse effect management [10,12]. However, real-world execution of these responsibilities is often constrained by high patient volumes, time limitations, commercial targets, and variability in regulatory enforcement [12,13]. Understanding how these systemic dynamics influence counseling quality and dispensing decisions is critical for developing structured clinical protocols.

### 1.5. Novelty and Rationale

Although the high prevalence of NSAID self-medication in Saudi Arabia is documented, significant gaps remain regarding how community pharmacists actively manage and document NSAID dispensing in real-world practice. Most prior regional studies have assessed consumer attitudes rather than the clinical oversight provided at the point of care. Furthermore, regional data from the Eastern Province, a key industrial and demographic hub with a diverse population and high community pharmacy density, are currently lacking. This study addresses these evidence gaps by evaluating real-world NSAID utilization patterns across major urban community pharmacies in the Eastern Province of Saudi Arabia. The findings will provide actionable baseline data to inform standardized counseling protocols, continuous professional development programs, and targeted health policy interventions aimed at promoting rational NSAID use.

## 2. Materials and Methods

### 2.1. Study Design and Setting

This study employed a direct observational cross-sectional design. It was conducted in 90 community pharmacies located in major urban centers of the Eastern Province of Saudi Arabia, including Al-Ahsa, Dammam, and Jubail Industrial City. The pharmacies were selected based on accessibility and willingness to participate, taking into consideration the study’s practical time and resource constraints. To improve geographic coverage and reduce concentration within a single location, pharmacies from multiple cities in the Eastern Province were included, such as Al-Ahsa, Al-Dammam, and Jubail Industrial City. The study reviewed pharmacies’ records of NSAIDs dispensed during the study period, including both prescription and over-the-counter (OTC) products.

### 2.2. Data Collection

Data were collected using a structured data collection sheet specifically developed for this study to ensure standardized documentation across participating sites. This exploratory, pharmacy-based observational study used convenience sampling, and the final sample size was determined by the number of eligible NSAID dispensing cases documented during the predefined study period across the participating pharmacies.

Data were recorded at the point of dispensing by trained pharmacy interns during regular working hours between 3 February and 16 March 2025. Both prescription-based and over-the-counter (OTC) NSAID dispensing encounters were included. Data were collected by reviewing pharmacy dispensing and prescription records; no patient interviews or direct interaction with patients were conducted at any stage of the study. The data collection form captured the following variables: demographic variables (age, gender, and body weight [kg]). NSAID medication data: drug name, dose (mg), dosage form, drug strength, frequency, duration of use, type of NSAID (selective or non-selective), number of pills dispensed, and NSAID use history within six months. Medication use and clinical information: indication for use, concurrent medications, total number of NSAIDs per prescription, and whether the medication was dispensed with or without a prescription. Dose appropriateness and documented contraindications were assessed based on the available prescription, dispensing information, and information provided by the dispensing pharmacist. The safety assessment was limited to evaluating potential clinically relevant drug–drug interactions. All documented concomitant medications were screened for interactions with the dispensed NSAID using a predefined interaction reference table developed based on UpToDate Lexidrug and the U.S. Food and Drug Administration (FDA) prescribing information. Concomitant medication use was assessed at the case level. For cases involving more than one concomitant medication, each medication was separately paired with the documented NSAID and screened for potential drug interactions. Therefore, interaction analyses were reported both at the case level (cases with at least one potential interaction) and at the medication-pair level (total NSAID–concomitant medication pairs assessed). The complete predefined interaction-reference table is provided in Supplementary Table S1.

### 2.3. Sample Size and Sampling Method

**Figure 1:**
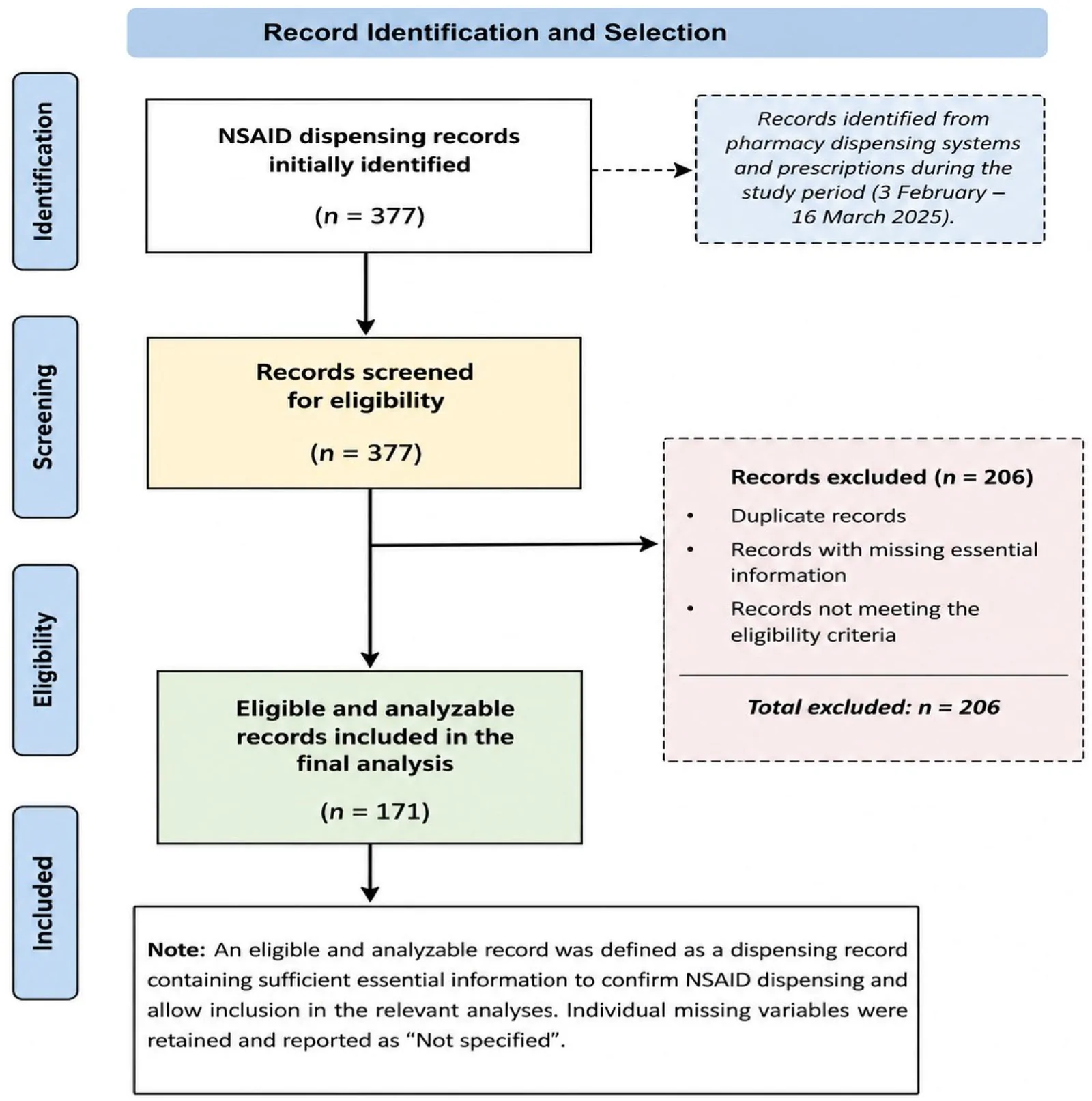
Flow diagram of record identification, screening, and inclusion.

A convenience sampling approach was employed. Community pharmacies were selected based on accessibility and willingness to participate, and all eligible NSAID dispensing encounters during the study period were consecutively recorded. Consequently, the sample was not intended to be statistically representative of all community pharmacies in the Eastern Province or Saudi Arabia. A total of 377 NSAID dispensing records were initially identified. After screening the records, 206 records were excluded because they did not meet the study eligibility criteria, including incomplete records, duplicate entries, or records with missing essential information required for analysis. A total of 171 eligible and analyzable records were included in the final analysis. Records with missing information for individual variables were retained when they met the eligibility criteria, and the missing information was reported as “Not specified” rather than excluding the entire record. The record identification, screening, exclusion, and final inclusion process is summarized in Figure 1.

### 2.4. Data Analysis

Data were analyzed using JMP^®^ Statistical Software version 18 (SAS Institute Inc., Cary, NC, USA) and R statistical software (version 2024.04.1). Descriptive statistics were used to summarize categorical variables as frequencies and percentages. Continuous variables were summarized using means and standard deviations where applicable. A significance level of *p* < 0.05 was predefined for statistical testing. To assess potential drug interactions, all documented concomitant medications were reviewed for a potentially clinically relevant interaction with the dispensed NSAID using a predefined interaction-reference table constructed using UpToDate Lexidrug and the FDA website [14,15]. Potential interactions were classified according to their expected clinical consequences which involve duplication of NSAID therapy, increased gastrointestinal ulceration or bleeding risk, renal impairment, reduced medication effectiveness, cardiovascular effects, and central nervous system effects. A dispensing case was classified as “potential interaction identified” when at least one documented concomitant medication had a potentially clinically relevant interaction with the dispensed NSAID. A case was classified as “no potential interaction identified” when none of the documented concomitant medications had an identified interaction.

Frequencies, percentages and 95% confidence intervals were calculated for the interaction groups.

Additional analyses were performed, such as Fisher’s exact tests for categorical comparisons, and logistic regression to assess associations between age group and prescription status. Statistical significance was set at *p*-value less than 0.05. Comparisons of prescription status across cities were conducted as exploratory analyses. Given the unequal number of observations across cities and clustering of encounters within pharmacies, city-level findings were interpreted cautiously.

### 2.5. Ethical Considerations

Ethical approval was obtained from the Institutional Review Board of King Faisal University (Ref. No. KFU-REC-2024-NOV-ETHICS2908). No personal identifiers (e.g., names, pharmacy record numbers, or contact information) were collected. Pharmacy interns were trained in study procedures and confidentiality requirements, and all data were recorded using anonymized case report forms and stored in a password-protected Microsoft Excel spreadsheet accessible only to the research team.

## 3. Results

### 3.1. Demographics

A total of 171 NSAID-related dispensing encounters were collected from community pharmacies in three cities within the Eastern Province of Saudi Arabia. The majority were recorded in Al-Ahsa (n = 93, 54.4%), followed by Dammam (n = 74, 43.3%), and Jubail (n = 4, 2.3%). Regarding gender distribution, females accounted for 93 cases (54.4%), while males represented 78 cases (45.6%).

### 3.2. NSAID Utilization Patterns

#### 3.2.1. Most Frequently Dispensed NSAIDs

**Figure 2:**
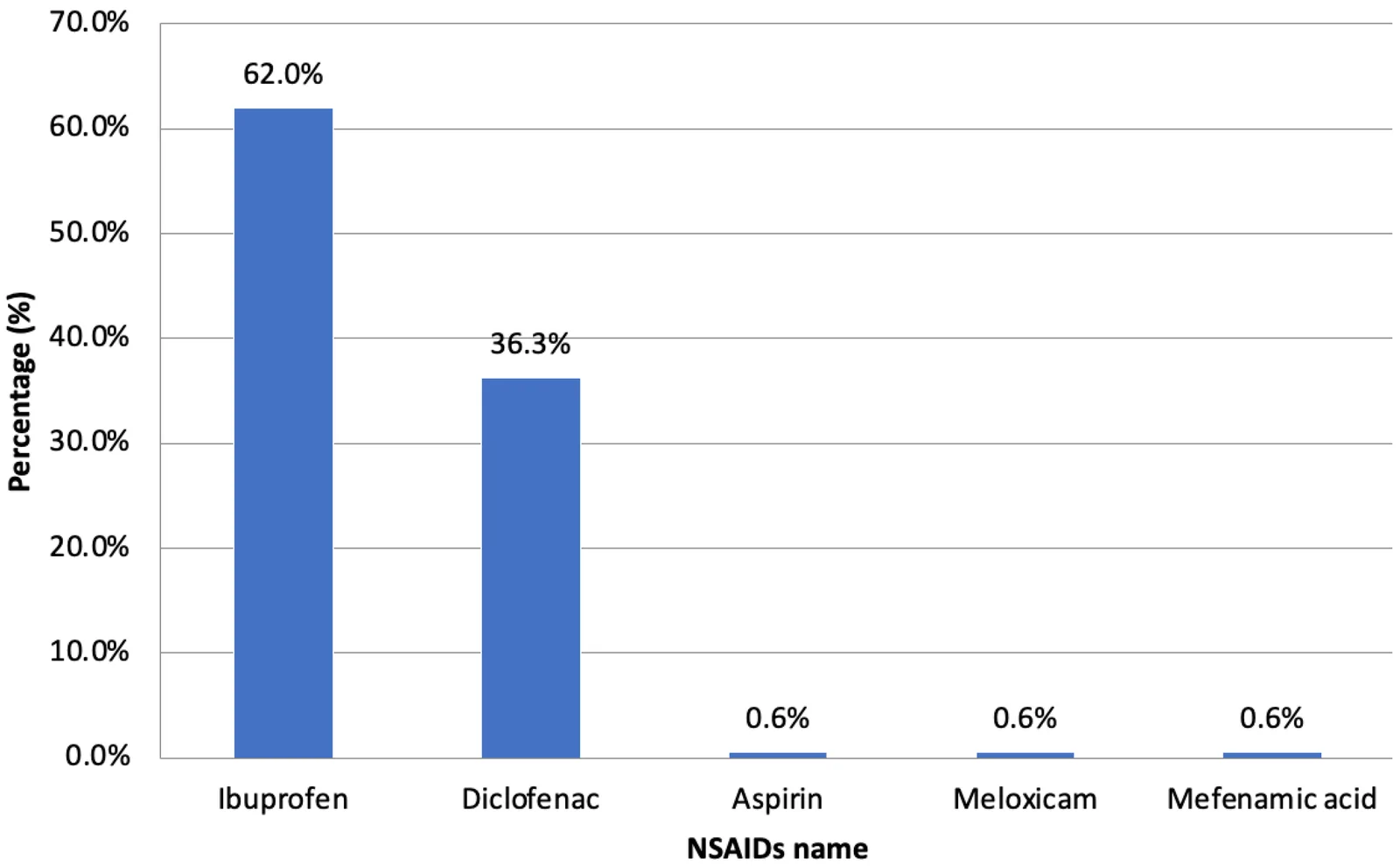
Most Frequently Dispensed NSAIDs.

Ibuprofen was the most frequently dispensed NSAID (n = 106, 62.0%), followed by diclofenac (n = 62, 36.3%). Other NSAIDs were rarely dispensed, including aspirin (n = 1, 0.6%), meloxicam (n = 1, 0.6%), and mefenamic acid (n = 1, 0.6%) (Table 1) (Figure 2).

#### 3.2.2. Frequency and Duration of Use

The most commonly documented dosing frequency was twice daily (n = 80, 46.8%), followed by once daily (n = 30, 17.5%), and three times daily (n = 29, 17.0%). Frequency was not specified in 27 cases (15.8%) (Table 2). Regarding duration of therapy, the most frequently reported duration was 5 days (n = 66, 38.6%), followed by 7 days (n = 26, 15.2%), 3 days (n = 15, 8.8%), and not specified in 41 cases (24%). Extended use of NSAIDs (≥10 days) was documented in 5 cases (2.9%) (Table 3).

#### 3.2.3. Recent NSAID Use History

Seventy-seven cases (45.0%) reported NSAID use within the previous six months. NSAID use history was not specified in 93 cases (54.4%), while only one case (0.6%) reported no prior use.

#### 3.2.4. Dosage Forms

The majority of NSAIDs were dispensed in tablet form (n = 131, 76.6%), followed by suspensions (n = 18, 10.5%), topical gels (n = 4, 2.3%), suppositories (n = 2, 1.2%), and capsules (n = 1, 0.6%). Dosage form was not specified in 15 cases (8.8%) (Table 4).

#### 3.2.5. Indications for NSAID Use

The most frequently documented indications for NSAID use were dental diseases and procedures (31.58%), followed by pain and inflammation (3.51%), respiratory tract infections (2.92%), musculoskeletal conditions (2.34%), and other medical indications (0.58%). However, the indication for NSAID use was not specified in 98 cases (57.3%) (Table 5).

#### 3.2.6. Prescription and Non-Prescription NSAID Dispensing Patterns:

Among the 171 documented NSAID dispensing cases, 98 (57.3%, 95% CI 49.8–64.5%) occurred without a prescription, whereas 73 (42.7%, 95% CI 35.5–50.2%) involved prescription-based dispensing. When comparing dispensation status across locations, prescription status varied significantly across the participating cities (Fisher–Freeman–Halton exact test, p<0.001), indicating that the distribution of prescription and non-prescription dispensing differed by location. In Al-Dammam, all 74 documented cases were dispensed without a prescription, whereas in Al-Ahsa, 73 of 93 cases (78.5%) involved a prescription and 20 (21.5%) occurred without a prescription. All four cases documented in Jubail occurred without a prescription. In the univariable logistic regression analysis, pediatric cases had higher odds of receiving an NSAID with a prescription compared with adult cases; however, the association was not statistically significant (OR 1.38, 95% CI 0.49–3.95, *p* = 0.536) (Table 6).

### 3.3. NSAIDs and Concomitant Medication Use 

Concurrent use of NSAIDs with other medications was documented in multiple cases. Frequently observed combinations included antibiotics (e.g., amoxicillin/clavulanic acid, metronidazole, cefixime, clindamycin), corticosteroids (e.g., dexamethasone), antihistamines (e.g., cetirizine, desloratadine), and muscle relaxants (e.g., tizanidine). The most common documented combination was amoxicillin/clavulanic acid with metronidazole (n = 11, 6.4%). Other combinations were observed at lower frequencies (Table 7 and Table 8). Concomitant medications were reported in 96.8% of cases in Al-Ahsa, 66.2% in Al-Dammam, and 50% in Jubail (Fisher’s exact test with simulated *p*-value, *p* < 0.001).

Of the 171 documented NSAID dispensing cases, 141 (82.5%, 95% CI 76.1–87.4%) involved at least one concomitant medication. Because several cases included multiple concomitant medications, a total of 219 NSAID–concomitant medication pairs were assessed for potential interactions. At least one potential interaction was identified in 11 (7.8%, 95% CI 4.4–13.4%) of the 141 cases with concomitant medication use, corresponding to 13 (5.9%, 95% CI 3.5–9.9%) potential interaction pairs.

## 4. Discussion

### 4.1. Key Findings

This study provides real-world insight into NSAID utilization patterns in community pharmacies across the Eastern Province of Saudi Arabia. The findings indicate that ibuprofen (62.0%) and diclofenac (36.3%) were the most frequently dispensed NSAIDs, with a predominance of oral tablet formulations (76.6%). NSAID use was generally short-term, most commonly 3–7 days, with dosing frequencies typically once or twice daily. Dental-related conditions were the most commonly documented indications; however, 57.3% of cases lacked recorded indications, and substantial gaps were also observed in dosing and duration data. Concurrent use with medications such as antibiotics and corticosteroids was identified. This study demonstrated that 57.3% of NSAID dispensing encounters occurred without a medical prescription, compared with 42.7% that involved a prescription. The overall analysis showed a higher proportion of non-prescription dispensing, although the difference was not statistically significant at the 0.05 level. The *p*-value of 0.066 suggests a possible trend toward more frequent non-prescription dispensing; however, these results should be interpreted cautiously. On the other hand, the location-level analysis showed a statistically significant association between city and prescription status. This suggests that the overall result may have important geographic differences in dispensing practices, and this prescription status differed significantly across cities (Fisher’s exact test, *p* < 0.001). This finding suggests that non-prescription access represented a considerable component of NSAID use in community pharmacies.

### 4.2. Interpretation of Utilization Patterns

The predominance of ibuprofen and diclofenac aligns with their widespread availability, affordability, and clinical familiarity, which have been consistently reported as key drivers of NSAID selection in both regional and global settings [16,17,18]. Ibuprofen, in particular, is widely regarded as a first-line NSAID for mild to moderate pain due to its favorable safety perception [18,19]. The predominance of short treatment durations (3–7 days) reflects use for acute pain conditions, particularly dental and musculoskeletal complaints, which is consistent with established prescribing practices [16,20]. The high reliance on tablet formulations further supports typical outpatient usage patterns. However, the limited diversity of NSAIDs and the high proportion of undocumented indications suggest potential constraints in prescribing variability and documentation practices. Similar gaps in structured documentation and risk assessment in community pharmacy settings have been previously reported [21].

### 4.3. Safety Considerations and Risk Behaviors

Although NSAIDs were predominantly used for short durations, several safety concerns emerge. The co-dispensing of NSAIDs with antibiotics and corticosteroids raises concerns regarding polypharmacy and potential drug–drug interactions, particularly in the absence of documented clinical assessment. NSAIDs are associated with well-established risks, including gastrointestinal bleeding, renal impairment, and cardiovascular events, particularly when used inappropriately or in high-risk populations [21]. Inadequate screening for these risks in community pharmacy settings has been previously highlighted as a significant concern [12]. While this study did not directly assess self-medication, patient decision-making, or prior medical advice, it documented a substantial proportion of NSAID dispensing without a prescription. Therefore, these findings should be interpreted specifically as evidence of non-prescription dispensing rather than as evidence of self-medication. Previous studies in Saudi Arabia have reported self-medication and NSAID use without medical consultation based on patient-reported data [16,22]; however, those findings cannot be directly inferred from the dispensing records analyzed in the present study. The documentation gaps observed in this study further support the likelihood of inadequate patient counseling and risk screening, and highlight the need for improved recording of clinical indications, concomitant medications, and relevant patient information during NSAID dispensing [23]. The comparisons between adult and pediatric cases showed no statistically significant differences in sex, prescription status, or concomitant medication use. These findings indicate that the key NSAID dispensing characteristics observed in this study were relatively consistent across age groups and were not driven exclusively by the much larger adult subgroup and may reflect broader pharmacy practice patterns within the participating sites. Additionally, in the univariable logistic regression analysis, pediatric cases had higher odds of receiving NSAIDs with a prescription than adult cases (OR 1.38, 95% CI 0.49–3.95), although this association did not reach statistical significance (*p* value = 0.536). The direction of the association may reflect more cautious prescribing and dispensing practices in pediatric patients. However, the limited number of pediatric cases reduced the precision of the estimate. Therefore, the finding should be regarded as a potentially meaningful pattern that warrants further investigation in larger and more balanced samples.

In our study, reported cases included at least one potential interaction identified in 11(7.8%, 95% CI 4.4–13.4%) of the 141 cases with concomitant medication use, corresponding to 13 (5.9%, 95% CI 3.5–9.9%) potential interaction pairs. This finding highlights the importance of reviewing concomitant medications before dispensing NSAIDs. This is because some of the concomitant medications observed in this study may increase the risk of gastrointestinal bleeding, kidney failure, or duplicate NSAID exposure. Nevertheless, these findings should not be interpreted as confirmed clinical harm but as evidence of potential interaction risk. This is because the study did not assess whether patients developed adverse reactions after dispensing of NSAIDs. Despite this limitation, the high frequency of potentially interacting combinations supports the need for routine medication reconciliation, pharmacist-led interaction screening, and patient counseling before NSAIDs are dispensed.

More than half of the documented NSAID dispensing encounters occurred without a prescription, indicating that non-prescription access remains common within community pharmacy practice. While the availability of over-the-counter NSAIDs facilitates timely management of acute pain and inflammatory conditions, it also places greater responsibility on community pharmacists to ensure appropriate medicine selection, assess contraindications and concomitant medications, and educate patients regarding dose limits, treatment duration, and potential adverse effects. The high frequency of non-prescription dispensing observed in this study reinforces the need to strengthen pharmacist-led risk assessment and patient counseling during routine NSAID dispensing.

### 4.4. Comparison with Previous Studies

The findings of the present study are generally consistent with previous studies conducted in Saudi Arabia, which have consistently identified ibuprofen as the most frequently used NSAID, followed by diclofenac. Asiri et al. reported a high prevalence of NSAID self-use among the Saudi population, with ibuprofen being the predominant agent because of its wide availability, perceived safety, and affordability [16]. Likewise, Almohammed et al. demonstrated that ibuprofen was the most commonly self-administered analgesic in the Eastern Province, accompanied by limited public awareness of its potential adverse effects [20]. More recently, Althemery et al. also reported sustained predominance of NSAID use, particularly ibuprofen, over the past decade in Saudi Arabia [17]. These findings collectively support the observed dispensing patterns in the present study.

Despite these similarities, important methodological differences should be considered when interpreting the findings. Most previous Saudi studies relied on questionnaire-based surveys assessing self-reported NSAID use, knowledge, or attitudes among members of the general public [16,20,22,24]. In contrast, the present study was based on direct documentation of NSAID dispensing encounters in community pharmacies, providing real-world information on medications actually dispensed rather than relying on participant recall. This approach reduces recall bias and allows characterization of dispensing practices, prescription status, concomitant medication use, and documentation quality at the point of care.

Another notable finding of the present study was the substantial proportion of undocumented clinical information, including indications, dosing details, and treatment duration. Although previous Saudi studies have primarily focused on estimating the prevalence of self-medication or evaluating public knowledge, relatively few have examined the completeness of pharmacy documentation during routine dispensing encounters. Therefore, our findings extend the existing Saudi literature by highlighting deficiencies in documentation and emphasizing the need to strengthen standardized dispensing procedures and pharmacist documentation practices in community pharmacy settings.

Internationally, similar utilization patterns have also been reported. In Spain, ibuprofen was the most frequently consumed NSAID [18], while studies from the United States likewise identified ibuprofen as the leading over-the-counter analgesic [19]. High rates of NSAID self-medication have also been documented in Italy [25], indicating that the widespread use of non-prescription NSAIDs represents a global phenomenon rather than one unique to Saudi Arabia.

Although our findings are generally consistent with previous reports from Saudi Arabia, they should be interpreted within the regional context of the Eastern Province. Differences in NSAID utilization patterns may exist across Saudi Arabia because of regional variations in population demographics, prevalence of chronic musculoskeletal disorders, healthcare accessibility, urbanization, socioeconomic characteristics, and community pharmacy practices. The Eastern Province is one of the country’s most densely populated and economically developed regions, with an extensive network of community pharmacies and a diverse population, factors that may influence medication-seeking behavior and dispensing practices. Consequently, while the present findings provide valuable insight into real-world NSAID dispensing in this region, they should not be assumed to represent national practice. Future multicenter studies involving different geographic regions of Saudi Arabia are needed to determine whether regional differences exist and to provide nationally representative estimates of NSAID utilization.

### 4.5. Implications for Practice and Policy

The high frequency of incomplete documentation observed in this study has important implications for patient safety and the quality of pharmaceutical care. Missing information regarding clinical indications, dosage, treatment duration, and concomitant medications may hinder pharmacists’ ability to evaluate the appropriateness of NSAID therapy, identify contraindications or potential drug–drug interactions, and provide individualized patient counseling. Inadequate documentation may also compromise continuity of care, particularly when patients obtain medications from different healthcare providers or community pharmacies. These findings highlight the need for standardized documentation practices and integrated electronic recording systems to support safer and more consistent NSAID dispensing.

The findings also reinforce the pivotal role of community pharmacists in promoting the safe and rational use of NSAIDs. Pharmacists are responsible for assessing patient suitability, identifying potential risk factors, and providing appropriate counseling regarding dosage, duration of therapy, and precautions associated with NSAID use [23]. However, previous studies from Saudi Arabia have reported variability in pharmacist counseling practices and risk assessment procedures [12,26]. Targeted continuing professional education, standardized dispensing protocols, and strengthened regulatory oversight may help improve the consistency and quality of pharmaceutical care while encouraging evidence-based NSAID dispensing practices.

In addition, the identification of potential drug–drug interactions among patients receiving concomitant medications underscores the importance of comprehensive medication review during NSAID dispensing. NSAIDs are known to interact with commonly prescribed medications, including antihypertensive agents, anticoagulants, antiplatelet drugs, corticosteroids, and selective serotonin reuptake inhibitors, potentially increasing the risk of bleeding, renal impairment, cardiovascular events, or reduced therapeutic efficacy. Although the present study identified potential rather than confirmed clinical interactions, these findings emphasize the importance of routinely reviewing patients’ medication histories and implementing appropriate risk-minimization strategies before dispensing NSAIDs.

### 4.6. Study Limitations

This study has several limitations that should be considered when interpreting the findings. First, the cross-sectional design precludes causal inferences regarding NSAID dispensing practices and captures prescribing and dispensing patterns at a single point in time. Consequently, temporal changes in dispensing behavior and their relationship with patient outcomes could not be assessed.

Second, a substantial proportion of the initially identified dispensing records were excluded because they were duplicates, incomplete, or lacked essential variables required for reliable analysis, such as the NSAID dispensed, prescription status, dosage information, or other key dispensing data. Although these exclusions were necessary to ensure data quality and analytical consistency, they may have introduced selection bias if the excluded records systematically differed from those included in the final analysis. In addition, incomplete documentation of clinical indications, dosing details, treatment duration, and previous NSAID use limited the comprehensiveness of the evaluation.

Third, the use of convenience sampling and recruitment from only three urban locations in the Eastern Province may have limited the representativeness of the study population. Furthermore, the unequal distribution of analyzed records across study sites, with most records originating from Al-Ahsa and relatively few from Jubail, may reflect differences in pharmacy participation, dispensing volume, or record availability rather than true regional variations in NSAID utilization. Therefore, the findings should be interpreted as representative of the participating pharmacies rather than all community pharmacies in the Eastern Province or Saudi Arabia, and caution is warranted when extrapolating the results to other settings.

Finally, the observational design restricted the scope of questions that could be addressed. The study could not determine whether dispensing practices influenced subsequent clinical outcomes, including medication adherence, adverse drug reactions, or healthcare utilization, nor could it evaluate the effectiveness of pharmacist counseling or interventions to improve NSAID use. Future research should therefore include prospective multicenter studies employing probability-based sampling, standardized electronic documentation systems, and balanced recruitment across geographic regions to improve representativeness and data completeness. In addition, cohort and quasi-experimental studies evaluating pharmacist-led educational interventions, standardized dispensing protocols, and clinical decision-support systems are needed to establish causal relationships and identify effective strategies for optimizing NSAID use in community pharmacy practice.

## 5. Conclusions

This study provides real-world evidence on NSAIDs dispensing practices in participating community pharmacies in the Eastern Province of Saudi Arabia. Ibuprofen and diclofenac were the most frequently dispensed NSAIDs, with oral tablets representing the predominant dosage form. More than half of the documented dispensing encounters occurred without a prescription, and substantial deficiencies were identified in the documentation of clinical indications, dosing information, and treatment duration. Potentially clinically relevant drug interactions were also identified among patients receiving concomitant medications.

These findings highlight opportunities to strengthen documentation practices, medication review, and pharmacist counseling during NSAID dispensing in community pharmacy settings. Given the study’s cross-sectional design and regional sampling approach, the findings should be interpreted within the context of the participating pharmacies and should not be generalized to all community pharmacy settings in Saudi Arabia. Future prospective multicenter studies are warranted to validate these findings and evaluate interventions aimed at improving the safe and rational dispensing of NSAIDs.

## Figures and Tables

**Table 1:** Most Frequently Dispensed NSAIDs.

NSAID Name	Count	Percentage (%)
**Ibuprofen**	106	62
**Diclofenac**	62	36.3
**Aspirin**	1	0.6
**Meloxicam**	1	0.6
**Mefenamic acid**	1	0.6

**Table 2:** Dosing Frequency of Dispensed NSAIDs.

Frequency	Count	Percentages (%)
**Twice daily**	80	46.8
**Once daily**	30	17.5
**Three times daily**	29	17.0
**Not specified**	27	15.8
**Other**	5	2.9

**Table 3:** Duration of NSAID Therapy.

Duration	Count	Percentage (%)
**1–2 months**	1	0.6
**1 day**	2	1.2
**1 month**	1	0.6
**2 days**	1	0.6
**3 days**	15	8.8
**4 days**	10	5.8
**5 days**	66	38.6
**5 days, after food**	1	0.6
**6 days**	1	0.6
**7 days**	26	15.2
**10 days**	2	1.2
**14 days**	3	1.8
**Not specific**	41	24
**PRN**	1	0.6

**Table 4:** Dosage Forms of Dispensed NSAIDs.

Dosage Form	Count	Percentage (%)
**Tablet**	131	76.6
**Suspension**	18	10.5
**Gel**	4	2.3
**Suppository**	2	1.2
**Capsule**	1	0.6
**Not specified**	15	8.8

**Table 5:** Documented Indications for NSAID Use.

Clinical Indication	Examples	Count, n (%)
**Unspecified indication**	Not specific	98 (57.3)
**Dental diseases and procedures**	Disease of pulp and periapical tissue; Periapical abscess without sinus; Dental inflamed pulp; Implant surgery; Embedded and impacted teeth; Retained dental root; Dental abscess; Swelling and abscess; Dental caries; Acute periodontitis; Gingivitis; Pocket and perio; Pain of right lower teeth; Pain of right upper teeth; Broken teeth	54 (31.58)
**Pain and inflammation**	Inflammation; Pain; Headache	6 (3.51)
**Respiratory tract infections**	Upper respiratory tract infection; Acute pharyngitis; Influenza	5 (2.92)
**Musculoskeletal conditions**	Arthritis; Back pain; Lumbago with sciatica; Neck pain	4 (2.34)
**Other medical conditions**	Primary hypertension	1 (0.58)

**Table 6:** Adult vs. Pediatric Cases Receiving NSAIDs with Prescription: A Logistic Regression Analysis.

Variable	β Coefficient	Standard Error	z Value	*p* Value	Odds Ratio	95% CI for OR
Intercept (Adult)	−0.325	0.163	−1.999	0.046	0.72	0.52–0.99
Pediatric vs. Adult	0.325	0.526	0.619	0.536	1.38	0.49–3.95

**Table 7:** Concurrent Medications Used with NSAIDs.

Medication Name	Count	Percentage (%)
**Amoxicillin/Clavulanic acid + Dexamethasone**	1	0.58
**Amoxicillin/Clavulanic acid + Metronidazole**	11	6.43
**Amoxicillin/Clavulanic acid + Metronidazole + Povidone-iodine**	1	0.58
**Amoxicillin/Clavulanic acid + Metronidazole + Paracetamol**	1	0.58
**Amoxicillin/Clavulanic acid + Paracetamol**	1	0.58
**Azithromycin**	1	0.58
**Azithromycin, Xylometazoline**	1	0.58
**Azithromycin + Tobramycin + Diclofenac**	1	0.58
**Cefdinir**	1	0.58
**Cefixime**	3	1.75
**Cefpodoxime**	1	0.58
**Cefuroxime**	1	0.58
**Cefuroxime + Cetirizine**	1	0.58
**Cefuroxime + Ambroxol**	1	0.58
**Cetirizine + Cefixime**	1	0.58
**Cetirizine**	1	0.58
**Chlorzoxazone + Paracetamol**	1	0.58
**Clindamycin**	3	1.75
**Desloratadine + Mometasone**	1	0.58
**Diclofenac + Tizanidine**	1	0.58

**Table 8:** Comparison of Adult vs. Pediatric Cases in Relation to Sex, Prescription Status, and Concomitant Use of Medications.

Characteristic	Pediatric	Adult	*p* Value
Total cases, n (%)	16	155	
**Sex**			
Male, n (%)	5 (31.2)	73 (47.1)	0.34
Female, n (%)	11 (68.8)	82 (52.9)	
**Prescription status**			
NSAID with prescription, n (%)	8 (50.0)	65 (41.9)	0.6005
NSAID without prescription, n (%)	8 (50.0)	90 (58.1)	
**Concomitant medication use**			
Concurrent medication use, n (%)	14 (87.5%)	127 (81.9%)	0.8
Without Concurrent medication use, n (%)	2 (12.5%)	28 (18.1%)	

Data are presented as n (%). Global *p*-values represent the overall association between age group and each categorical variable. Pearson’s chi-square test was used when expected cell counts were adequate; Fisher’s exact test was used when expected cell counts were small. A *p*-value < 0.05 was considered statistically significant.

## Data Availability

The original contributions presented in this study are included in the article/Supplementary Material. Further inquiries can be directed to the corresponding author.

## Supplementary Materials

The following supporting information can be downloaded at: https://www.mdpi.com/article/10.3390/pharmacy14050123/s1, Supplementary Table S1. NSAID–concomitant medication interaction reference table.

## References

[1] McGettigan P., Henry D. (2011). Cardiovascular risk and inhibition of cyclooxygenase: A systematic review of the observational studies of selective and nonselective inhibitors of cyclooxygenase 2. PLoS Med..

[2] Derry S., Wiffen P.J., Moore R.A. (2017). Relative efficacy of oral analgesics in acute postoperative pain. Cochrane Database Syst. Rev..

[3] Sostres C., Gargallo C.J., Lanas A. (2014). Nonsteroidal anti-inflammatory drugs and upper gastrointestinal bleeding. Best Pract. Res. Clin. Gastroenterol..

[4] Lanas A., Chan F.K.L. (2017). Peptic ulcer disease. Lancet.

[5] Harirforoosh S., Asghar W., Jamali F. (2013). Adverse effects of nonsteroidal anti-inflammatory drugs: An update of gastrointestinal, cardiovascular and renal complications. J. Pharm. Pharm. Sci..

[6] Bhala N., Emberson J., Merhi A., Abramson S., Arber N., Baron J.A., Bombardier C., Cannon C., Farkouh M.E., Baigent C. (2013). Vascular and upper gastrointestinal effects of non-steroidal anti-inflammatory drugs: Meta-analyses of individual participant data from randomised trials. Lancet.

[7] Montuori P., Shojaeian S.Z., Pennino F., D’aNgelo D., Sorrentino M., Di Sarno S., Nubi R., Nardo A., Triassi M. (2024). Consumer awareness and knowledge regarding use of non-steroidal anti-inflammatory drugs (NSAIDs) in a metropolitan area. Front. Pharmacol..

[8] Albsoul-Younes A., Tahaineh L., Al-Sawalha N. (2021). Assessment of public knowledge, attitudes, and practices regarding non-steroidal anti-inflammatory drugs. Int. J. Clin. Pract..

[9] Barakat H.E., Aziz C.N., Abougalambou S.S.I. (2023). Evaluation of the knowledge, practices, and attitudes of community pharmacists towards adverse effects of non-steroidal anti-inflammatory drugs (NSAIDs): A cross-sectional study. J. Pharm. Policy Pract..

[10] Showande S.J., Akinbode T.E. (2024). Nonsteroidal anti-inflammatory drug use by patients: Impact of modular educational training on pharmacists’ questioning, counselling and risk assessments. Explor. Res. Clin. Soc. Pharm..

[11] Alharbi A.O., Almjayishi S.A., Aldarwish R.I., Almeshari M.A., Aljumydi M.T., Faraj R.M. (2023). Patient-provider communication regarding NSAID risks among patients with rheumatic disorders in Saudi Arabia. Saudi Pharm. J..

[12] Malebari A., Khayyat A.N., Mahdali R.A., Alamoudi J.S., Alsayed B.Y., Alrasheed S.A. (2020). Evaluation of the community pharmacists’ performance in the screening of non-steroidal anti-inflammatory drugs risks in Saudi Arabia. Saudi Med. J..

[13] Shareef J., Sridhar S., Al Naqbi S.H., Bakshi A.I. (2025). Beyond pain relief: A cross-sectional study on NSAID prescribing, polypharmacy, and drug interaction risks in community pharmacies. Healthcare.

[14] Connor R.F. (2026). UpToDate Drug Interactions. UpToDate. Wolters Kluwer.

[15] U.S. Food and Drug Administration Drugs@FDA: FDA-Approved Drugs.

[16] Asiri O., Alzahrani A., Alshehri K., Althomali O., Alameen A., Serwah M. (2020). Prevalence of non-steroidal anti-inflammatory drugs usage and assessment of knowledge related to its complications among Saudi population; a cross-sectional study. Int. J. Med. Dev. Ctries..

[17] Althemery A.U., Alsubaie M., Alharbi H., Alqahtani A., Alsubaie G., Alotaibi A. (2025). Patterns of Opioid and NSAID Use in Saudi Arabia from 2010 to 2020. J. Psychoact. Drugs.

[18] Marcos Gragera R., Kogevinas M. (2018). Epidemiology of non-steroidal anti-inflammatory drugs consumption in Spain. The MCC-Spain study. BMC Public Health.

[19] Wilcox C.M., Cryer B., Triadafilopoulos G. (2005). Patterns of use and public perception of over-the-counter pain relievers: Focus on nonsteroidal antiinflammatory drugs. J. Rheumatol..

[20] Almohammed B.A. (2023). Frequency and knowledge of analgesics self-use and their adverse effects in the Eastern Province of Saudi Arabia. Cureus.

[21] Kasciuškevičiūtė S., Gumbrevičius G., Vendzelytė A., Sčiupokas A., Petrikonis K., Kadusevicius E. (2018). Impact of the World Health Organization pain treatment guidelines and the European medicines agency safety recommendations on nonsteroidal anti-inflammatory drug use in Lithuania: An observational study. Medicina.

[22] Magadmi R.M., Kamel F.O., Hagras M.M., Alhmied H.I., Aljumaiy W.H., Saqat D.F., Magadmi M.M. (2022). Knowledge and attitudes regarding the self-use of pain medications in Saudi Arabia: A cross-sectional study. J. Microsc. Ultrastruct..

[23] Kampamba M., Mulenga P., Mudenda S., Chabalenge B., Zulu J., Chimombe T., Mufwambi W., Ngula M.I., Hamachila A., Hikaambo C.N.A. (2024). Roles of Community Pharmacists in Screening and Disseminating of Information about Non-Steroidal Anti-Inflammatory Drugs Risks: Implications for Drug Safety Assessment. Pharmacol. Pharm..

[24] Alhur A., Alhur A., Alfayiz A., Alotaibi A., Hansh B., Ghasib N., Alharbi F., Albalawi N., Aljohani A., Almaghthawi A. (2023). Patterns and prevalence of self-medication in Saudi Arabia: Insights from a nationwide survey. Cureus.

[25] Garofalo L., Di Giuseppe G., Angelillo I.F. (2015). Self-medication practices among parents in Italy. BioMed Res. Int..

[26] Al Qarni H., Alrahbini T., AlQarni A.M., Alqarni A. (2020). Community pharmacist counselling practices in the Bisha health directorate, Saudi Arabia–simulated patient visits. BMC Health Serv. Res..

